# Posterior cruciate ligament repair with suture tape augmentation: a case series with minimum 2-year follow-up

**DOI:** 10.1186/s40634-021-00337-y

**Published:** 2021-04-15

**Authors:** Graeme P. Hopper, Ahmer Irfan, Joanne M. Jenkins, William T. Wilson, Gordon M. Mackay

**Affiliations:** 1 College, of Medical, Veterinary and Life Sciences, University of Glasgow, University Avenue, Glasgow, G12 8QQ Scotland; 2 University of Alabama, Tuscaloosa, USA; 3 NHS Greater Glasgow & Clyde, Glasgow, UK; 4 University of Strathclyde, Glasgow, UK; 5 University of Stirling, Stirling, UK

**Keywords:** Knee, PCL, PCL Rupture, PCL Repair

## Abstract

**Purpose:**

The posterior cruciate ligament (PCL) is an important stabilizer of the knee and can be damaged in up to 20% of ligamentous injuries. Numerous techniques for surgical treatment have been described in the literature with none shown to be clearly superior. The aim of this study was to assess the 2-year outcomes of PCL repair with suture tape augmentation.

**Methods:**

Seventeen patients undergoing PCL repair with suture tape augmentation were prospectively followed up for a minimum of two years. One patient was lost to follow-up leaving sixteen patients in the final analysis (94.1%). Indications for this procedure were acute Grade III PCL ruptures, symptomatic chronic tears and PCL tears as part of a multi-ligament injury. Exclusion criteria were patients with retracted PCL remnants or poor tissue quality. Patient-reported outcomes were measured using the Knee Injury and Osteoarthritis Outcome Score (KOOS), Western Ontario and McMaster Universities Osteoarthritis Index (WOMAC), Visual Analogue Pain Scale (VAS-pain), Veterans RAND 12 Item Health Survey (VR-12) and Marx Activity Scale. Patients with any postoperative complications were identified. Mean differences between the outcomes pre-operatively and at two years postoperatively were evaluated using paired t-tests with significance set at *p* < 0.05.

**Results:**

The mean KOOS at 2 years was 87.0, 75.5, 93.0, 69.6 and 54.2 for pain, symptoms, ADL, sport/recreation and QOL respectively. These improved significantly from 60.2, 49.8, 65.0, 33.0 and 34.2 preoperatively (*p* < 0.05). The mean WOMAC scores at 2 years were 91.0, 78.3 and 93.0 for pain, stiffness and function respectively. These improved significantly from 63.0, 51.7 and 65.0 preoperatively (*p* < 0.01). The VAS score improved from 3.0 to 0.8 (*p* < 0.01) and the VR-12 score improved from 34.9 to 50.9 at 2 years (*p* < 0.001). However, the Marx activity scale decreased from 8.7 pre-injury to 6.3 at 2 years (N.S.). One patient (6.3%) suffered a re-rupture.

**Conclusion:**

PCL repair with suture tape augmentation demonstrates satisfactory patient reported outcome measures at minimum 2-year follow-up. These figures compare favorably with success rates described in the literature for PCL reconstruction techniques. Therefore, PCL repair with suture tape augmentation is an effective treatment option in selected patients.

**Level of evidence:**

IV

## Introduction

Posterior cruciate ligament (PCL) injuries are commonly caused by pre-tibial trauma, hyperflexion, and hyperextension of the knee [3]. Although isolated PCL injuries are more common in athletes, many present with associated ligament injuries [1, 2]. Acute injuries present with knee pain and swelling; with the posterior drawer test most commonly used for clinical diagnosis. In general, grade III injuries (≥10 mm posterior instability) or those with combined collateral ligament injuries are considered for operative management [4].Numerous techniques have been described in the literature for the surgical management of patients with PCL ruptures [1, 2, 5, 11, 24]. Historically, primary PCL repair was the most common surgical option; however, PCL reconstruction procedures are now more commonly performed, following the trend of managing injuries to the anterior cruciate ligament (ACL).

Internal bracing of the PCL with suture tape augmentation, similar to ACL internal bracing, reinforces the ligament and acts as a secondary stabilizer [13]. This protects the ligament during the healing phase allowing natural healing whilst permitting early mobilization and accelerated rehabilitation. Additionally, the need for graft harvest is avoided, removing the potential morbidity associated with this. Moreover, the proprioceptive properties that are retained in the native PCL could also contribute to improved functional recovery and return to sporting activity. Long term, this may have a beneficial effect in preventing the development of early onset posttraumatic osteoarthritis.

The aim of this study is to report on the clinical outcomes of the cohort at minimum 2 years following PCL repair with suture tape augmentation. We hypothesized that there would be satisfactory patient-reported outcome measures at 2 years postoperatively.

## Methods

### Patient selection

Approval to conduct this study was sought from the local medical ethics committee (RH030518). Between February 2013 and August 2017, 17 patients with a PCL rupture underwent PCL repair with suture tape augmentation. Indications for this procedure were acute grade III PCL tears, symptomatic chronic tears and PCL tears as part of a multi-ligament injury. Exclusion criteria were patients with retracted PCL remnants or poor tissue quality, who would require a conventional PCL reconstruction. All surgical procedures were performed by a single surgeon.

One patient was lost to follow-up leaving 16 patients in the final analysis (94.1%). With respect to injury patterns, 5 patients (31.3%) presented with multi-ligament injury (1 underwent ACL and PCL repair and 4 underwent PCL repair and posterolateral corner repair) and 11 patients (68.7%) presented with isolated PCL injuries. The mean age at the time of surgery was 37 ± 11 years (range, 19-57) and all the patients were male. Mean follow-up was 48 ± 11 months (range, 24-66 months).

### Surgical technique

Standard anteromedial and anterolateral portals are used with the addition of an accessory posteromedial portal. The first step is to elevate the PCL and track it down to its tibial insertion. The residual PCL fibers are retained and pushed posteriorly with the other posterior structures allowing for a safe and adequate exposure. An anteromedial incision is made over the proximal tibia and a standard PCL guide is used to drill a 3.5mm tunnel. The drill is advanced under direct vision to minimize the risk of complication. The anterior tibial cortex is tapped and the drill is switched for a FiberStick™ (Arthrex, Naples, FL). The FiberWire^®^ (Arthrex, Naples, FL) is grasped out of the FiberStick™ and taken through the anteromedial portal.

The insertion point of the PCL on the femur is then identified and marked using electrosurgery to ensure accuracy when the guide pin is passed. Reaming allows easier passage of the femoral button (Retrobutton^®^ or TightRope RT^®^, loaded with FiberTape^®^, Arthrex, Naples, FL) when it is shuttled from the anterolateral portal directly through the tunnel. The suture tape is then secured 1cm distal to the tibial tunnel using a 4.75mm SwiveLock^®^ (Arthrex, Naples, FL) with the knee in 90 degrees of flexion and an assistant providing anterior translation (maximum anterior drawer) to hold the tibia in a reduced position with adequate tension on the PCL. Prior to insertion the laser line is marked which indicates the anatomical length of the PCL. If there are any reservations the knee is put through a full range of motion in the reduced position prior to marking, as excessive tensioning can result in difficulty achieving full extension. Securing the suture tape distally is an essential step as this restores the length of the anatomical PCL. Additionally, prior to cutting the suture tape, anteroposterior stability is re-checked to confirm reduction and elimination of posterior sagging.

Patients are allowed to fully weight bear with crutches as required during the first two weeks after surgery. The perceived limited pain and swelling of this procedure in comparison to other techniques allows accelerated early phase rehabilitation with a focus on early range of motion and restoration of function. No external bracing is required. Most patients will return to pivoting sports around 5-6 months following surgery when neuromuscular function has recovered. No changes were made to the rehabilitation regime for those with multi-ligament injuries which were repaired simultaneously [7].

### Clinical and functional evaluation

Patients were reviewed in the outpatient clinic until 6 months postoperatively, where stability and range of motion was assessed.

After informed consent was given by the patients, they were evaluated prospectively with the Surgical Outcome System (SOS, Arthrex, Naples, Fl, USA). SOS is a web-based tool which sends questionnaires and patient-reported outcome measures (PROMs) at scheduled timepoints. Prior to introducing the SOS system and analysing the prospective follow-up data, permission was sought from the local medical ethics committee.

The PROMs measured are the Knee Injury and Osteoarthritis Outcome Score (KOOS) [18], Western Ontario and McMaster Universities Osteoarthritis Index (WOMAC) [15], Visual Analogue Pain Scale (VAS) [3], Veterans RAND 12 Item Health Survey (VR-12) [19], and Marx Activity Scale [14]. In addition to these PROMs, all patients were asked about their overall satisfaction at 2-years postoperatively. This was subdivided into 4 categories: pain, movement, function, and sports. All the patients were also contacted by email/telephone at the time of this analysis to collect data about any complications.

### Data analysis

KOOS subscales, WOMAC subscales, VAS-pain, VR-12 subscales and Marx activity scales were presented as means with standard deviations and ranges over the course of the two-year follow up. Data normality was assessed using the Shapiro-Wilk test, and once confirmed, mean differences between the outcomes pre-operatively and at two years postoperatively evaluated using paired t-tests with significance set at *p*<0.05. As this was a single cohort study with no control group, a power calculation was not used to guide the study design. All analyses were performed with SPSS 21.0 (IBM, New York, USA).

## Results

All patients were found to have a stable knee on manual clinical examination using a posterior drawer test when reviewed in the outpatient clinic at 6 months postoperatively by the senior author. PROMs data was then used to assess the longer-term outcomes of these patients.

**Fig. 1:**
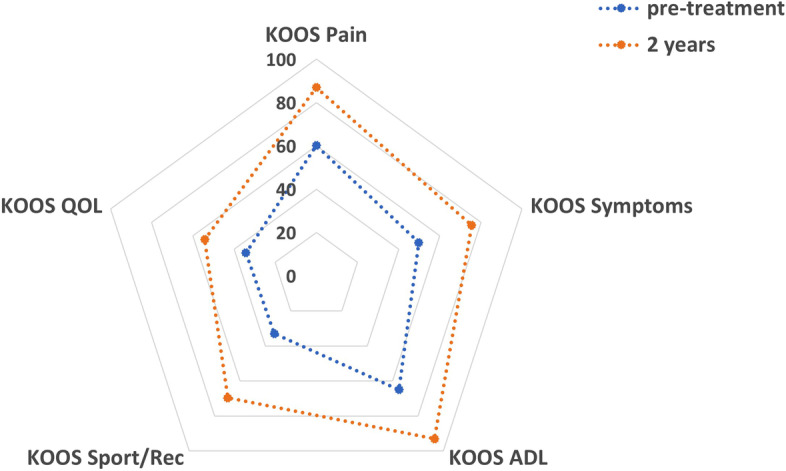
Spider chart demonstrating improvements at 2-year follow-up (orange line) in all sub-sections of the KOOS in comparison to the preoperative score (blue line)

**Fig. 2:**
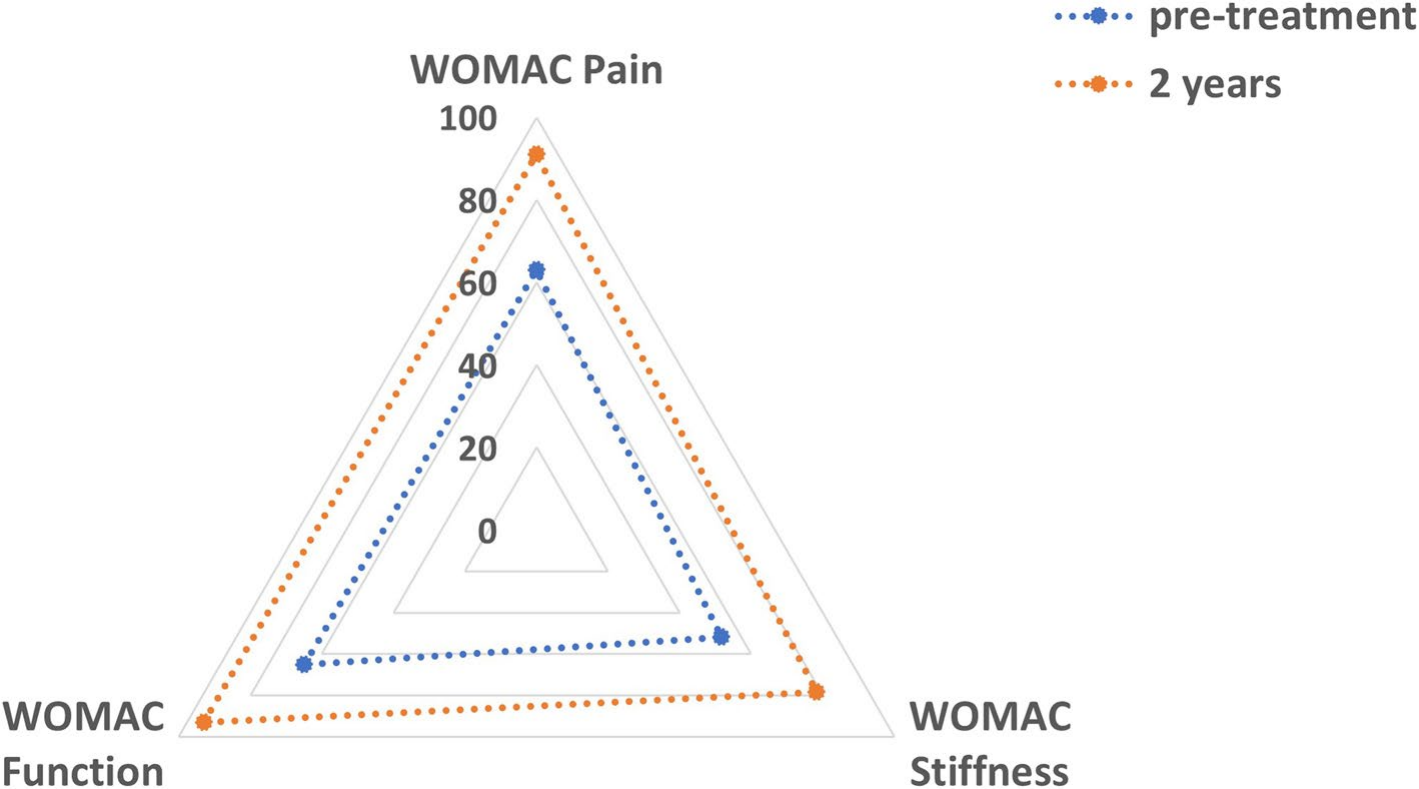
Spider chart demonstrating improvements at 2-year follow-up (orange line) in all sub-sections of the WOMAC score in comparison to the preoperative score (blue line)

The mean KOOS at 2 years was 87.0, 75.5, 93.0, 69.6 and 54.2 for pain, symptoms, ADL, sport/recreation and QOL respectively. These improved significantly from 60.2, 49.8, 65.0, 33.0 and 34.2 preoperatively (*p*<0.05) (Fig. 1). The mean WOMAC scores at 2 years were 91.0, 78.3 and 93.0 for pain, stiffness and function respectively. These improved significantly from 63.0, 51.7 and 65.0 preoperatively (*p*<0.01) (Fig. 2).

**Fig. 3:**
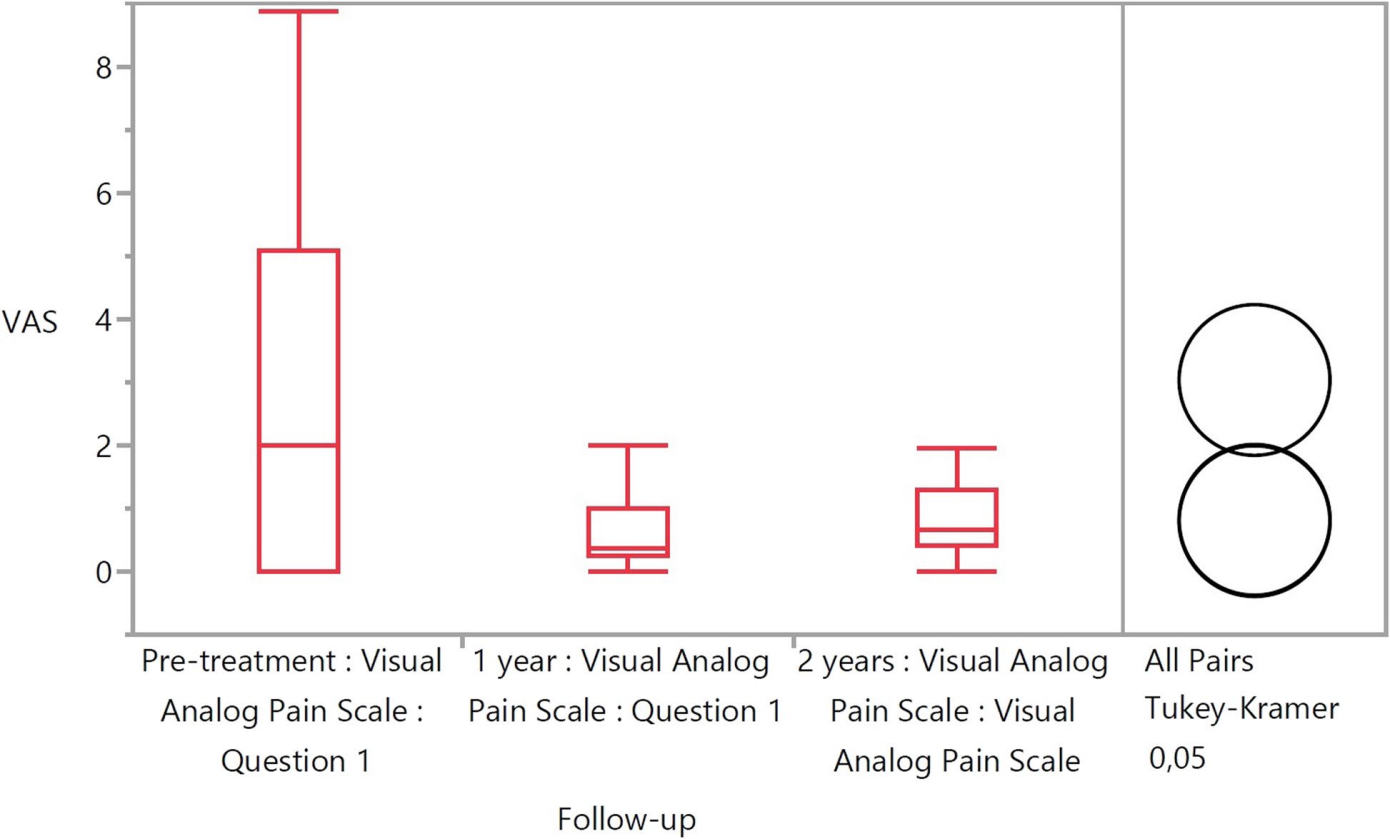
Chart demonstrating the VAS-pain scores at the different time intervals

**Fig. 4:**
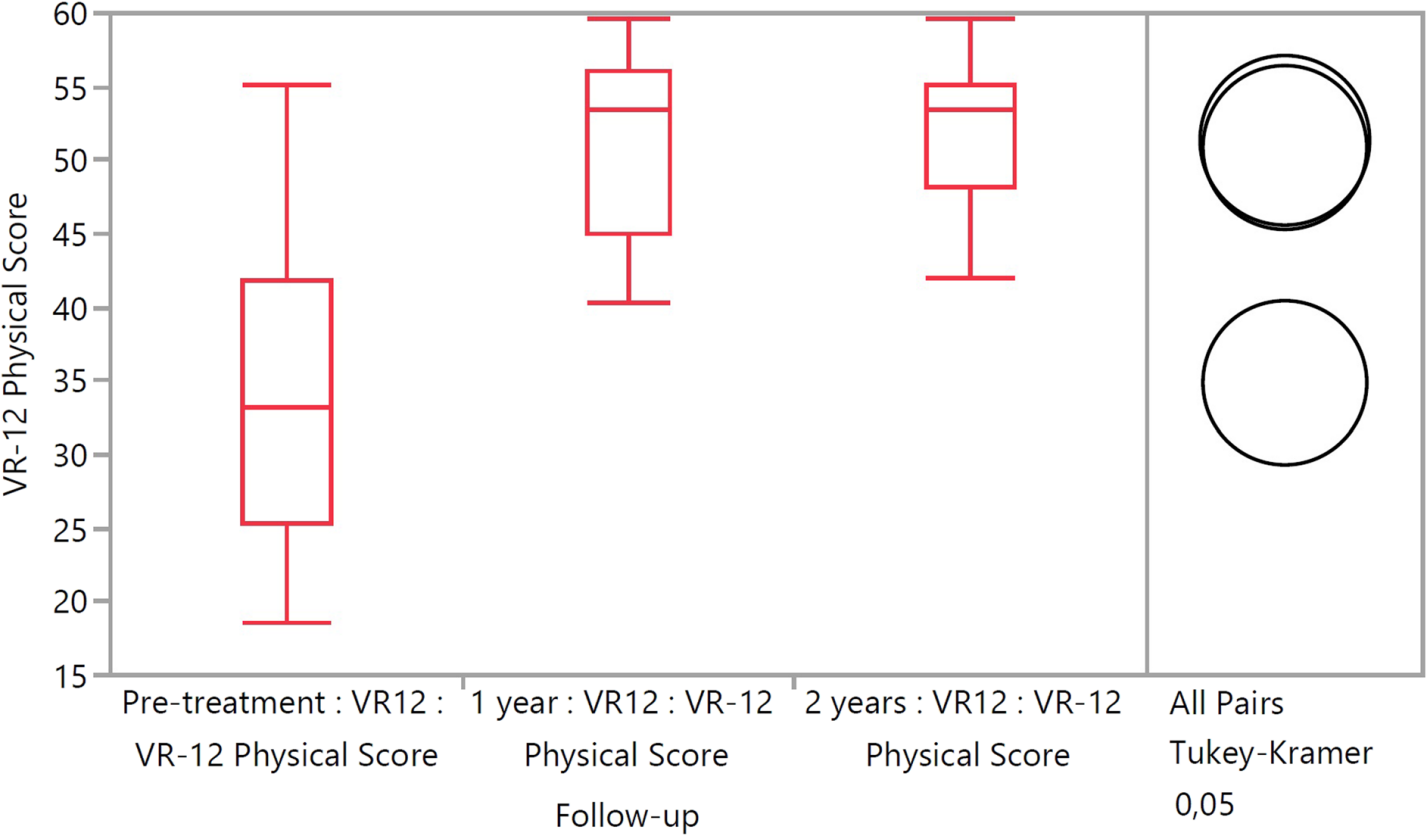
Chart demonstrating the VR-12 physical scores at the different time intervals

The VAS score improved from 3.0 to 0.8 (*p*<0.01) (Fig. 3) and the VR-12 score improved from 34.9 to 50.9 at 2 years (*p*<0.001) (Fig. 4).

There was a decrease seen in the Marx activity scale, from 8.7 pre-injury to 6.3 at 2 years (N.S.).

The majority of patients were satisfied with the outcome of their PCL repair with suture tape augmentation at 2-year follow-up. 81.3% of patients felt the surgery exceeded or met their expectations with regards to reducing pain and improving movement and 87.5% with regards to resuming their normal functions of daily living. 68.8% of patients felt the surgery exceeded or met their expectations with regards to resuming normal sporting activities (Table 1).

**Table 1:** Overall satisfaction of patients at 2-year follow-up

	**Pain** **(% patients)**	**Movement** **(% patients)**	**Function** **(% patients)**	**Sports** **(% patients)**
Exceeded expectations	37.5	31.3	31.3	18.8
Met expectations	43.8	50	56.2	50
Did not meet expectations	18.7	18.7	12.5	31.2

### Complications

Further surgery on the same knee was required in 2 patients (12.5%). One patient underwent microfracture for a new osteochondral injury 2 years postoperatively. The other patient suffered from a re-rupture and underwent a PCL reconstruction using allograft 3 years following initial surgery. No other complications or further knee surgeries were reported. There were no significant demographic or outcome differences between patients requiring re-operation and the other patients in the cohort.

## Discussion

The main finding in this study was the encouraging 2-year follow-up results of this novel technique of PCL repair with suture tape augmentation. There were significant improvements in all aspects of the KOOS and WOMAC scores as well as a significant reduction in the VAS for pain and a significant increase in the VR-12 physical score. There was a decrease in the Marx score postoperatively, though this was not statistically significant. Although this decrease in the Marx score has not been studied following PCL repairs, this has been reported following ACL repair and ACL reconstruction [6, 20]. In our cohort, only 1 patient (6.3%) suffered from a re-rupture and subsequently underwent a PCL reconstruction with no complications reported thereafter.

Suture tape augmentation reinforces the ligament, acts as a secondary stabilizer and protects the ligament during the healing phase which allows natural healing and early mobilization. Furthermore, the morbidity associated with graft harvest is avoided leading to a reduction in muscle atrophy postoperatively, thereby accelerating rehabilitation. Moreover, the proprioceptive properties that are retained in the native PCL could benefit long-term recovery and return to sporting activity [13]. Additionally, the tunnels associated with this PCL repair technique are situated in the same position as the larger tunnels used for autografts or allografts in PCL reconstruction. As a result, the revision surgery is without the additional complexity often associated with revision following PCL reconstruction [12]. There was no evidence of synovitis, erosions or cyst formation on further imaging or at the time of revision surgery. This addresses a major concern and highlights the difference between the internal bracing technique used in this study and traditional synthetic grafts [23].

PCL repair was originally performed as an open procedure with inconsistent results [8, 17, 21]. More recently, arthroscopic PCL repair has been described using a number of different techniques. These have shown promise, with satisfactory patient reported outcomes following repair after PCL soft tissue avulsion injuries [25]. A small case series utilizing suture anchors to repair PCL soft tissue peel-off injuries also reported satisfactory outcomes [5]. There has also been the description of a technique similar to ours, with PCL repair and augmentation with an internal brace [24]. Furthermore, Otto et al. [16] described their results of internal bracing of acute PCL lesions with minimum follow-up of 12 months but they were only able to include 14 of 27 patients. Nonetheless, there are no clinical outcome results of arthroscopic PCL repair with suture tape augmentation with 2-year follow-up in the literature.

The results we have described show significantly better outcomes than historical papers investigating PCL repairs. Hughston et al. [8] found good objective and subjective results in 65 and 90% of patients respectively. However, all patients in their population presented with multi-ligament injury with only 55% of patients having proximal tears. Posterior knee instability is a common problem postoperatively with it being reported in 100% of patients in one study [17]. Although this may not be inherently linked to subjective outcomes, with half of patients in a study having posterior instability, despite 81% of patients reporting good or excellent subjective outcomes [21].

As PCL reconstruction techniques are more commonly performed, several clinical outcomes studies have been published. A systematic review comparing single-bundle versus double-bundle PCL reconstruction conveyed significantly improved posterior stability and International Knee Documentation Committee (IKDC) scores in the double-bundle group [2]. Another systematic review compared PCL reconstruction with allograft versus autograft and demonstrated improved clinical outcomes in each group with no differences between the groups [1]. A review comparing PCL reconstruction to PCL augmentation found comparable results in each group [4]. The augmentation procedures analyzed included a remnant posterior cruciate ligament-augmenting stent procedure and double-bundle augmentation with Achilles allograft [10, 26].

Our results are more comparable to the studies following conventional PCL reconstruction techniques [1, 2, 4]. However, comparison can be difficult as these studies utilised different measures for patient reported outcomes. They used the IKDC Score [9], Lysholm and Tegner scores [22] while we did not use any of these patient-reported outcome measures.

In those patients that have undergone PCL reconstruction, the mean age was 29.7 years [2] compared to a mean age of 37 years in our population. There are several theories to explain this discrepancy. Firstly, injury patterns may be different between these two groups. For an injury to be amenable to PCL repair with suture tape augmentation, the ligament remnant needs to be of good quality and non-retracted. This is more common with proximal PCL ruptures and may be a more common occurrence in an older population. Postoperatively, older patients may not put the extra demand on the PCL compared to a younger patient and this may be reflected in the outcomes. However, the Marx activity scores indicate that the patients in this cohort are physically active.

There are several limitations associated with this study, including the lack of clinical testing at 2 years and the absence of radiological assessment. In addition, the majority of our outcome scores capture pre-operative rather than pre-rupture data. Subsequently, it can be difficult to analyze how close to previous normal function patients are postoperatively, as is the goal of the repair. Moreover, a number of our patients have torn their PCL as part of a multi-ligament injury. Although this is similar to the figures described in historic PCL literature and it is known that most PCL injuries are associated with multi-ligament injuries to the knee. Nonetheless, we did not find any differences between the isolated PCL group and the multi-ligament group. In addition, no direct comparisons can be made to PCL reconstruction procedures as there was no randomization and all of the patients within the inclusion criteria underwent PCL repair with suture tape augmentation. We also have a small patient cohort and a more powered study would allow us to make stronger conclusions. Many studies involving PCL injuries report on relatively small patient numbers, largely due to the low incidence of PCL rupture compared to ACL rupture.

This study indicates that PCL repair with suture tape augmentation is an acceptable alternative operative treatment to conventional PCL reconstruction techniques in appropriate cases. However, further clinical studies are necessary with larger patient numbers and longer follow-up as well as randomized studies to further assess these encouraging early results.

## Conclusion

This is the first case series that describes minimum 2-year follow-up results of patients with a PCL injury treated with a suture tape augmentation repair technique. There was a significant improvement in patient reported outcome measures at 2 years postoperatively. This shows that PCL repair with this technique is an effective treatment option in suitable patients.
